# Therapeutic Potential for Cannabidiol on Alzheimer’s Disease-Related Neuroinflammation: A Systematic Review and Meta-Analysis

**DOI:** 10.3390/ijms262411963

**Published:** 2025-12-11

**Authors:** Shuo Wu, Tracia Rajiah, Afia B. Ali

**Affiliations:** UCL School of Pharmacy, 29-39 Brunswick Square, London WC1N 1AX, UK; shuo.wu.24@ucl.ac.uk (S.W.); tracia.rajiah.21@alumni.ucl.ac.uk (T.R.)

**Keywords:** cannabidiol, Alzheimer’s disease, neuroinflammation, neurodegeneration, endocannabinoid system, meta-analysis

## Abstract

Alzheimer’s disease (AD) is a pervasive neurodegenerative disorder characterized by chronic neuroinflammation; current interventions primarily offer symptomatic relief. Cannabidiol (CBD), a non-psychoactive phytocannabinoid, exhibits multi-target therapeutic potential due to its established anti-inflammatory and neuroprotective properties. While growing interest exists, the evidence regarding CBD’s effects on AD-related neuroinflammation has not been robustly consolidated in a quantitative meta-analysis. Therefore, this article reviews the current literature around CBD related to its potential in alleviating neuroinflammation, followed by a meta-analysis of preclinical and clinical studies using random-effects modeling to assess CBD efficacy on neuroinflammation and clinical outcomes in AD. In preclinical AD models, the meta-analysis demonstrated that CBD significantly and consistently reduced key markers of neuroinflammation and reactive gliosis, specifically glial fibrillary acidic protein (GFAP) (*p* < 0.0001), Interleukin-6 (IL-6), and inducible nitric oxide synthase (iNOS). Effects on other markers, such as tumor necrosis factor-alpha (TNF-α) and interleukin-1 beta (IL-1β), were non-significant and heterogeneous. Clinical evidence, though limited by small sample size and heterogeneity, showed a borderline significant benefit favoring CBD for overall behavioral symptoms (*p* = 0.05), agitation, and caregiver distress. Adverse events were typically mild. We conclude that CBD demonstrates biologically consistent anti-inflammatory efficacy in preclinical AD models. While current clinical data remains insufficient to substantiate efficacy, they suggest promising signals for behavioral control. Determining CBD’s full therapeutic potential in AD necessitates future rigorous, mechanism-driven trials with standardized preparations and biomarker-anchored endpoints.

## 1. Introduction

### 1.1. Background

Alzheimer’s disease (AD) is the most prevalent neurodegenerative disease as of 2020, affecting approximately 50 million people globally. The clinical profile is characterized by progressive cognitive deficits (short- and long-term memory impairment) alongside neuropsychiatric symptoms (e.g., behavioral problems) and, eventually, profound motor and communication difficulties [1,2]. The resulting care burden is immense; for example, the annual cost in the UK is projected to rise from GBP 42 billion in 2024 to nearly GBP 90 billion by 2040 [3]. This escalating socio-economic crisis is compounded by the inadequate efficacy of current treatments, which fail to target a key underlying pathology: chronic neuroinflammation [4,5,6,7,8,9,10,11,12,13,14,15]. Consequently, there is an urgent need to identify new therapeutic agents that modulate this inflammatory pathway. Cannabidiol (CBD), a non-psychoactive Phytocannabinoid with demonstrated anti-inflammatory and neuroprotective properties, has emerged as a compelling candidate for this investigation.

The classical pathophysiology of AD centers on two hallmark lesions: amyloid beta plaques (Aβ) and neurofibrillary tangles. Aβ deposition initiates when the sequential cleavage of amyloid precursor protein (APP) by β-and γ-secretases generates insoluble amyloid beta monomers. These monomers subsequently aggregate to form Aβ plaques. Consequently, these plaques disrupt neuronal signaling, leading to characteristic cognitive deficits, including confusion and memory impairment [2]. Neurofibrillary tangles develop following the hyperphosphorylation of tau, a key protein responsible for maintaining the neuronal cytoskeleton and microtubules. This pathological event detaches tau from the microtubules, leading to the formation of toxic intracellular aggregates that ultimately trigger neuronal apoptosis [2].

Chronic neuroinflammation is now recognized as a major mechanism driving the severity and progression of AD, actively contributing to Aβ and tau pathologies [16,17]. This inflammatory response exacerbates neurodegeneration by triggering the disruption of perineuronal nets, altering gamma-aminobutyric acid type A receptor (GABAA) receptor subtype expression, and leading to the degeneration of inhibitory interneurons [16,17,18,19]. In 1990, several studies explored the relationship between anti-inflammatory treatments and long-term non-steroidal anti-inflammatory drugs with AD; results showed that AD’s risk decreased by 50% [20,21,22]. This supports the claim that neuroinflammation plays a key role in AD’s development.

**Figure 1:**
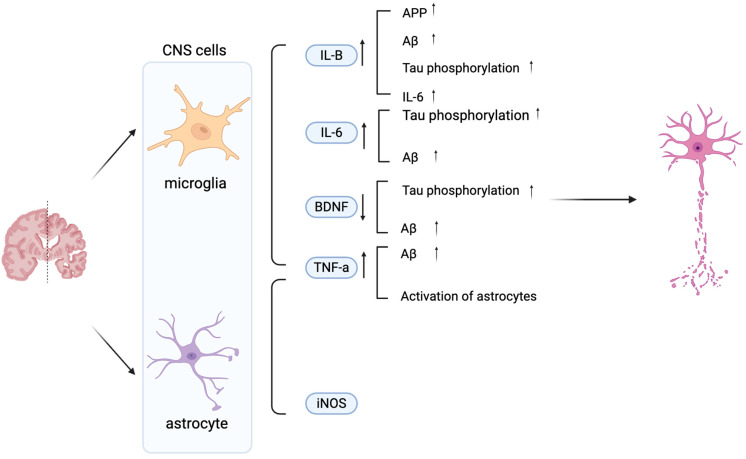
Schematic diagram illustrating the effects of microglia- and astrocyte-derived mediators on neuronal dysfunction in AD. Key 1. IL-6, TNF-α, iNOS, and BDNF modulate AD pathology by promoting Aβ accumulation, Tau phosphorylation, and neuronal damage. Increased IL-1β, IL-6, TNF-α, and iNOS exacerbate neuroinflammation, while decreased BDNF contributes to synaptic loss and impaired neuronal survival. Vertical arrows denote increased or decreased expression levels, while horizontal arrows indicate the direction of regulatory interactions between cells. Created in BioRender. Shuo, S. (2025) https://BioRender.com/wzzu9gq.

Aβ and tau pathologies initiate an inflammatory signaling cascade, primarily via the nuclear factor-kappa B (NF-κB) pathway, which drives the sustained activation of microglia and astrocytes and triggers the release of pro-inflammatory mediators [9]. Microglia activity can be measured via the protein marker, ionized calcium-binding adaptor molecule 1 (Iba1), and indirectly via cytokine levels. The three main cytokines in AD released by microglia are interleukin-1 beta (IL-1β), tumor necrosis factor-alpha (TNF-α), and Interleukin-6 (IL-6). These can have various effects on AD, which can additionally be achieved via Brain-derived neurotrophic factor (BDNF) as shown in Figure 1 [2,15,23,24,25]. On the one hand, these cytokines can also be released by astrocytes. Activated astrocytes can be measured via glial fibrillary acidic protein (GFAP), an astrocytic cytoskeleton intermediate filament protein, and inducible nitric oxide synthase (iNOS) [26,27,28,29] (Figure 1). Collectively, the activation of both microglia and astrocytes contributes significantly to neuroinflammation in AD through the synergistic release of these cytokines and the production of neurotoxic nitric oxide catalyzed by iNOS. This chronic inflammation exacerbates AD progression. This insight may enable the development of anti-inflammatory approaches that may slow AD progression.

Currently, treatments are based on inhibiting N-methyl-D-aspartate (NMDA) receptors and acetylcholinesterase. The premise of the NMDA receptor inhibitors as a therapeutic target is related to blocking the excessive glutamate released at synapses during AD, which will activate NMDA receptors, consequently causing an influx of Ca^2+,^ leading to neuronal dysfunction and apoptosis [30]. Common inhibitors include rivastigmine, donepezil, galantamine, and memantine. The rationale for using acetylcholinesterase inhibitors, including donepezil, rivastigmine, and galantamine, is based on the complex alterations of cholinergic function and cognitive impairment in AD [1,2,30]. Both of these treatments, however, carry many side effects and are typically provided in conjunction with psychiatric pharmacotherapy to aid efficacy [31].

**Figure 2:**
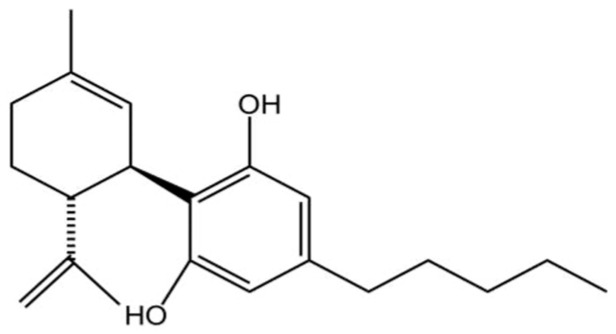
Chemical structure of CBD(C_21_H_30_O_2_). Created in BioRender. Shuo, S. (2025) https://BioRender.com/wtl3bp5.

Given the limited efficacy and symptomatic focus of current AD treatments, there is an urgent need to develop novel therapeutic agents that address the underlying pathology. CBD, a non-psychoactive phytocannabinoid with the molecular formula C_21_H_30_O_2_ (Figure 2), This molecule is becoming increasingly popular in the research field related to neurodegenerative conditions such as multiple sclerosis, epilepsy, AD, and Tourette’s syndrome [32] in modulating the disease pathology [33].

The chemical structure of CBD confers a variety of biological activities. The aromatic ring enhances the stability of the molecule and has significant antioxidant properties that help reduce inflammation and protect cells from oxidative stress-induced damage [34]. Its bicyclic structure affects its ability to bind to receptors and indirectly regulates cannabinoid receptor type 1 (CB1) and cannabinoid receptor type 2 (CB2) receptor signaling pathways, which are involved in the regulation of physiological functions such as mood, appetite, and pain perception [35]. The hydroxyl group contained in CBD is a potent scavenger of free radicals, which reduces oxidative stress-induced inflammation and cellular damage, and is particularly important for reducing neurological damage and enhancing neuroprotection [36]. Its isopropyl side chain enhances the hydrophobicity of the molecule, thereby increasing its ability to penetrate cell membranes and its bioavailability in the body [37]. This hydrophobicity also allows CBD to cross the blood–brain barrier (BBB) and act directly on the brain and central nervous system [38]. It is precisely owing to these distinct structural features that CBD can establish intricate and multifaceted biological connections with the ubiquitously expressed endogenous cannabinoid system (ECS), a complex lipid signaling network fundamental to maintaining physiological homeostasis [39,40,41,42,43,44,45].

### 1.2. Anti-Inflammatory Mechanisms of CBD

The ECS is a central physiological system whose structural features and interactions with phytocannabinoids such as CBD and Δ9-tetrahydrocannabinol (THC) have been recently highlighted, revealing significant therapeutic potential [46,47,48,49]. The ECS consists of three core components: endogenous cannabinoids, cannabinoid receptors, and metabolic enzymes. Endocannabinoids are self-synthesized lipid-based signaling molecules, primarily anandamide (AEA) and 2-arachidonoylglycerol (2-AG), which regulate mood, appetite, pain perception, and immune responses [50,51]. Although differing in mechanisms and metabolic pathways, AEA and 2-AG share functional similarities with phytocannabinoids by activating the same cannabinoid receptors [52]. These effects are mediated via the two major cannabinoid receptors, CB1 and CB2, which are differentially distributed throughout the body [53]. CB1 receptors are predominantly expressed in the brain and central nervous system, influencing mood, memory, pain, and appetite, while CB2 receptors are mainly found in immune tissues such as the spleen and immune cells, modulating inflammatory responses and immune function [51,52,53,54,55,56]. Metabolic enzymes maintain ECS homeostasis by regulating the synthesis and degradation of endocannabinoids [57]. Fatty acid amide hydrolase (FAAH) degrades AEA, thereby influencing mood, appetite, and stress responses, while monoacylglycerol lipase (MAGL) primarily hydrolyses 2-AG, affecting anti-inflammatory and analgesic processes [56,57]. The enzymatic activity of FAAH and MAGL determines the duration and intensity of cannabinoid signaling via CB1 and CB2 receptors, underscoring their importance in ECS-mediated physiological regulation [55,58].

**Figure 3:**
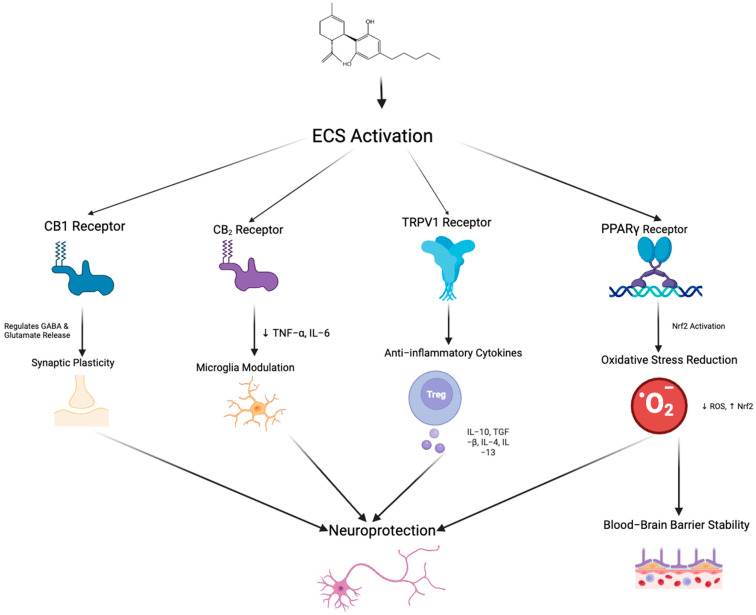
Schematic representation of the potential neuroprotective mechanisms mediated by endocannabinoid system activation through multiple receptor pathways. Arrows indicate the direction of regulatory interactions among receptors, signaling pathways, and cellular responses. Activation of CB1 receptors regulates GABA and glutamate release, contributing to synaptic plasticity. CB2 receptor stimulation reduces pro-inflammatory cytokines (TNF-α, IL-6) and modulates microglial activity. TRPV1 receptor activation promotes the release of anti-inflammatory cytokines (e.g., IL-10, TGF-β, IL-4, IL-13) through the involvement of regulatory T cells. Peroxisome proliferator-activated receptor gamma receptor activation triggers Nrf2 signaling, which decreases reactive oxygen species and enhances resistance to oxidative stress, ultimately leading to improved blood–brain barrier stability. Collectively, these pathways converge to promote neuroprotection via synaptic modulation, immune regulation, and maintenance of BBB integrity. Created in BioRender. Shuo, S. (2025) https://BioRender.com/xoc2kta.

The “on-demand” function of the ECS is a key feature, meaning the synthesis and release of endogenous cannabinoids such as AEA and 2-AG is not continuous but triggered under specific physiological or pathological conditions [58]. Endocannabinoid synthesis occurs rapidly in response to neuronal depolarization, inflammatory stimuli, stress, increased intracellular calcium, or activation of other G-protein-coupled receptors [59]. Unlike conventional neurotransmitters, endocannabinoids are not stored in vesicles but are synthesized from membrane phospholipid precursors via enzymes such as N-acyl-phosphatidylethanolamine-specific phospholipase D and Diacylglycerol lipase and released immediately into the extracellular space [60]. Their actions are typically localized, modulating synaptic activity by binding to CB1 or CB2, for example, inhibiting glutamate or gamma-aminobutyric acid release from presynaptic neurons [59]. This signaling is rapidly terminated by enzymatic degradation, primarily through FAAH and MAGL [58]. Under pathological conditions such as neuroinflammation or tissue injury, ECS activity is upregulated to exert neuroprotective effects, reducing excitotoxicity via CB1 or regulating immune responses and inflammation via CB2 [61]. This dynamic and localized regulation enables the ECS to rapidly adapt to environmental changes and maintain physiological homeostasis.CBD exerts its neuroprotective and anti-inflammatory effects through diverse mechanisms that target both the ECS (Figure 3) and non-cannabinoid pathways.

CBD primarily modulates the ECS by enhancing the activity of its endogenous ligands. This is achieved through dual actions: enzyme inhibition and transporter interference. Specifically, CBD inhibits the activity of FAAH, the key enzyme responsible for degrading AEA, thereby increasing in vivo levels of AEA and prolonging its activation of CB1 receptors. [62]. Simultaneously, CBD enhances the bioavailability of 2-AG by inhibiting MAGL activity, promoting the sustained activation of both CB1 and CB2 receptors [63,64]. Furthermore, CBD blocks the intracellular transport of AEA by competitively binding to fatty acid binding proteins (FABPs) [65], an interference that synergizes with FAAH inhibition to stabilize AEA signaling and potentiate its neuromodulatory effects on both CB1 and CB2 receptors [65,66]. CBD acts as a negative conformational modulator of the CB1 receptor, diminishing the binding capacity of exogenous agonists while enhancing the efficacy of endogenous ligands [67]. This bidirectional mechanism optimizes the dynamic regulation of synaptic transmission and neuronal excitability. In addition, ECS regulation by CBD is region-specific [67,68]. In the central nervous system, it increases AEA to modulate CB1 for mood and pain control [66,67,68,69,70], while in peripheral tissues, it raises 2-AG to activate CB2, producing anti-inflammatory and immunomodulatory effects.

Beyond the ECS, CBD’s neuroprotective actions also stem from significant activity via non-cannabinoid receptors and associated pathways, which greatly expand its therapeutic scope. CBD directly curbs microglial activation by downregulating the toll-like receptor 4 (TLR4)/NF-κB signaling pathway, thereby reducing the expression of pro-inflammatory cytokines. Notably, it activates transient receptor potential vanilloid 1 (TRPV1), a cation channel involved in temperature sensing, chemical stimuli, and inflammation [68]. TRPV1 activation modulates pain perception, reduces inflammation, and induces local desensitization, beneficial for chronic pain and inflammatory diseases, including neuropathic pain. Additionally, CBD partially activates the 5-hydroxytryptamine receptor 1A, crucial for mood, stress, and social behavior regulation, producing significant anxiolytic and antidepressant effects [69]. This action synergizes with AEA-mediated CB1 receptor activity, enabling cross-system modulation.

CBD also activates peroxisome proliferator-activated receptors (PPARs), a class of nuclear receptors involved in lipid metabolism, inflammation control, and cellular energy regulation [70]. Through activation of PPARα and PPARγ, CBD regulates metabolic homeostasis and inflammatory responses in cells [70]. PPARγ activation reduces pro-inflammatory cytokines, alleviating tissue damage [71], while in metabolic diseases, CBD improves lipid metabolism and insulin sensitivity [72]. CBD also modulates G protein-coupled receptors, inhibits cancer cell migration, and activates TRPA1, enhancing anti-inflammatory and analgesic effects [73,74].

In summary, CBD works together to maintain ECS homeostasis through a multi-mechanistic pathway of enzyme inhibition, transporter regulation, receptor modification, and cross-system receptor action. By synergistically enhancing the activity of the endocannabinoid system and acting on multiple non-cannabinoid targets, CBD can address complex diseases such as chronic pain, anxiety, inflammation, and metabolic disorders. Future research should focus on translational studies to optimize its clinical application in multiple disease settings.

### 1.3. Aims of the Meta-Analysis

Currently, research on CBD in attenuating neuroinflammation associated with AD remains limited. Up to now, there are no meta-analyses on this topic, which also hinders the translation of preclinical research results to clinical applications. This study aims to fill this gap by integrating data from animal models and clinical studies through a systematic review and meta-analysis to assess the efficacy of CBD in reducing neuroinflammation in AD. A systematic search of PubMed and Embase databases was conducted to analyze the effects of CBD on neuroinflammatory indicators and to provide directions for future research. Based on the existing preclinical and clinical literature [1,2,3,4,5,6,7,8,9,10,11,12,13,14,15,16,17,18,19,20,21,22,23,24,25,26,27,28,29,30,31,32,33,34,35,36,37,38,39,40,41,42,43,44,45,46,47,48,49,50,51,52,53,54,55,56,57,58,59,60,61,62,63,64,65,66,67,68,69,70,71,72,73,74,75] and the urgent need for more effective AD treatments, we hypothesize that CBD administration will significantly reduce markers of neuroinflammation in animal models of AD and has the potential to translate into clinical benefits for AD patients.

## 2. Methodology

### 2.1. Search Strategy and Information Sources

This systematic review and meta-analysis were conducted in accordance with the Preferred Reporting Items for Systematic Reviews and Meta-Analyses (PRISMA) 2020 guidelines [76]. A comprehensive literature search was performed in PubMed and EMBASE databases to ensure inclusion of peer-reviewed biomedical and pharmacological research. PubMed ensures extensive indexing of life sciences and neuroscience journals, whilst EMBASE provides broader access to pharmacology and toxicology research, particularly covering European literature. This dual-database strategy maximizes search sensitivity and specificity whilst maintaining methodological rigor and minimizing duplicate retrieval, in accordance with PRISMA guidelines.

This study focuses on AD, the most prevalent neurodegenerative disorder globally. The core pathophysiological features of AD—including neuroinflammation, oxidative stress, and β-amyloid deposition—align closely with the potential pharmacological mechanisms of CBD [77]. The focus on AD aims to enhance study homogeneity and improve the reliability of pooled effect estimates.

The search period encompassed studies published between 1978 and 2024. The year 1978 was chosen as the starting point, as it marked the inception of cannabinoid neuropharmacology research, when Mechoulam et al. proposed that cannabinoids may exert effects through specific neural mechanisms rather than nonspecific lipid interactions [78]. The year 2024 was selected as the cutoff date to incorporate the most current experimental and clinical evidence available at the time of this review’s publication.

The search strategy included the following keywords and their synonyms or expanded terms: “CBD”, “neuroinflammation”, “inflammation”, “inflammatory markers”, “A”, “animal study”, “clinical”, and “in vivo”.

### 2.2. Study Selection

For Preclinical Studies, titles and abstracts were screened independently to identify relevant preclinical research investigating CBD in AD models. Eligible studies included in vivo animal models with either Aβ-induced pathology or transgenic AD mice, where CBD was administered, and outcomes on neuroinflammatory markers were reported. Exclusion criteria were in vitro studies, use of THC or other drug combinations, and studies targeting diseases other than AD. References of included studies were also manually screened for additional eligible articles.

Titles, abstracts, and full texts of clinical trials were reviewed to identify studies that assessed the effects of CBD or CBD-containing preparations on patients with AD or dementia. Only clinical trials that reported outcomes such as behavioral symptoms, agitation, indicators of neuroinflammation, or burden of care were included. Given the limited number of clinical studies in this area, this review also included relevant studies with healthy volunteers in clinical studies. However, studies of other neurologic conditions or observational studies without interventions were not included.

### 2.3. Data Extraction and Outcomes

From each eligible preclinical study, the following data were extracted: study title, author, year, study design, animal model, sample size, age, AD pathology, CBD dose and route of administration, treatment duration, outcome measures, and main findings. Primary outcomes focused on the effects of CBD on neuroinflammatory cytokines, enzymes, astrocytic protein, microglial protein, and neurotrophic factor. Where statistical data were not reported, mean values were estimated from bar graphs, and standard deviations were calculated from error bars.

For each clinical trial, extracted variables included study title, author, year, study design, country, number of participants, treatment duration, CBD dose and formulation, comparator (placebo or mixed THC: CBD extract), clinical endpoints assessed, and main findings. Primary outcomes included behavioral symptoms, agitation, and caregiver distress, while secondary outcomes included safety, adverse events, and mechanistic readouts where available.

### 2.4. Risk of Bias Assessment

Risk of bias in animal studies was assessed using SYRCLE’s Risk of Bias (RoB) tool, due to its provision of a customized framework for preclinical animal research [79]. Ten domains were evaluated: sequence generation, baseline characteristics, allocation concealment, random housing, blinding of caregivers, random outcome assessment, blinding of outcome assessment, incomplete outcome data, selective outcome reporting, and other biases. Each item was rated as low, high, or unclear risk of bias.

The Cochrane RoB 2.0 tool, the established gold standard for evaluating risk of bias in randomized clinical trials, was applied to assess the methodological quality of clinical studies [79]. Key domains included randomization process, deviations from intended interventions, missing outcome data, measurement of outcomes, selective reporting, and overall risk of bias. Each study was rated as low risk, some concerns, or high risk of bias.

### 2.5. Ethics

All included preclinical studies reported compliance with institutional and national ethical standards for animal experimentation. Clinical trials were approved by appropriate research ethics committees, and all participants provided informed consent. As this work is a secondary analysis of published data, no additional ethical approval was required.

### 2.6. Statistical Analysis

Meta-analyses were conducted using Review Manager (RevMan) version 5.4. Continuous outcomes were summarized as standardized mean differences (SMDs) with 95% confidence intervals (CI). A random-effects model was employed due to expected heterogeneity across studies. Statistical significance was set at *p* < 0.05. Heterogeneity was assessed using the I^2^ statistic, with values above 50% considered indicative of substantial heterogeneity. Subgroup analyses (e.g., acute vs. chronic CBD treatment, different dosing regimens) were performed where data permitted, to explore sources of heterogeneity.

## 3. Summary of Included Studies

### 3.1. Preclinical Models

**Figure 4:**
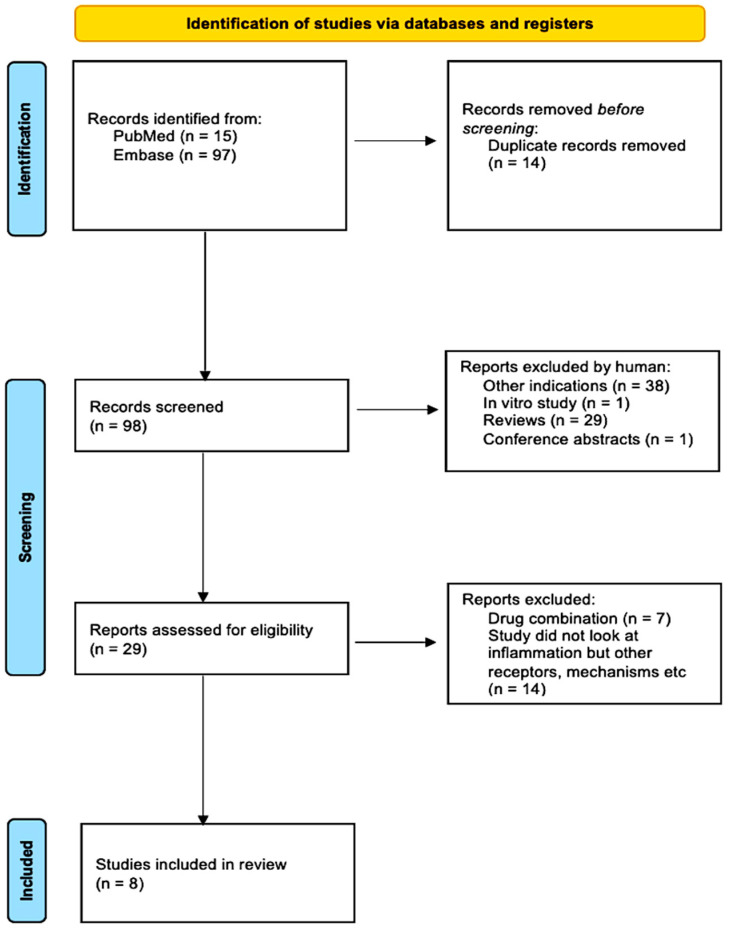
PRISMA flow chart summarizing the study selection process from 112 studies to 8 studies.

Following the search strategy, 112 studies were yielded from the databases EMBASE and PubMed. In total, 14 duplicated studies were removed via EndNote, leaving 98. Investigated other indications or conducted in vitro experiments were removed, consequently leaving 29 studies. The remaining studies were then thoroughly inspected; those that examined THC or other drug combinations or investigated inflammation were removed, leaving the records to be screened. After reading titles, abstracts, reviews, conference papers, and studies, I finally selected 8 studies that fit the criteria. Figure 4 summarizes this process via a PRISMA flow chart.

The 8 selected studies were based on 5 different countries—Italy, Australia, China, Spain, and the USA. Most trials conducted a cross-sectional study, except for one study by David Cheng, in which a longitudinal study was conducted. Amongst these trials, all used mouse models were used, 4 of which were inoculated with Aβ peptide and 4 transgenic [71,72,80,81,82,83,84,85,86,87]. All studies varied in CBD dose from 2.5 to 30 mg/kg; on the one hand, the duration of the study also varied from 7 days to 8 months. Most studies, to different degrees and mechanisms, showed that neuroinflammation decreased when given CBD. Table 1 summarizes this.

### 3.2. Clinical Trials

A total of 72 records were retrieved from the EMBASE and PubMed databases according to the same search strategy as for clinical studies. After removing 13 duplicates through EndNote, 59 studies remained. Subsequently, the literature on other indications, in vitro experiments, or reviews was excluded, and nine full-text studies were retained for detailed assessment. These nine studies were further screened to exclude literature that did not study AD alone. Only five studies that met the criteria were included after reading the full title, abstract, review, conference paper, and study content.

A total of five clinical trials were included, originating from multicenter teams in the UK, Israel, and Italy. The predominant design was the randomized, double-blind, Randomized Controlled Trial (RCT). Among the studies, the studies involved different doses of CBD medication given, while others used both CBD medication given alone and CBD medication treatment containing THC, and the studies ranged from 6 weeks to 12 weeks. All studies were conducted in clinical use and contained control groups of patients with AD and healthy volunteers. The studies evaluated the pharmacological effects of CBD by different metrics and scoring judgment mechanisms and showed that the anti-inflammatory mechanism of CBD may work by reducing IL-6 & TNF-α-mediated microglial activation and may enhance the patients’ quality of life. Table 2 summarizes this.

## 4. Meta-Analysis

We conducted a meta-analysis to synthesize preclinical and clinical evidence on CBD and neuroinflammation in AD and psychiatric disorders. Pooled datasets (Table 1 and Table 2) were analyzed in Review Manager 5.4 using continuous outcomes to compute standardized mean differences with 95% confidence intervals; two-sided *p* values < 0.05 were considered statistically significant.

### 4.1. Risk of Bias: Preclinical Studies

**Figure 5:**
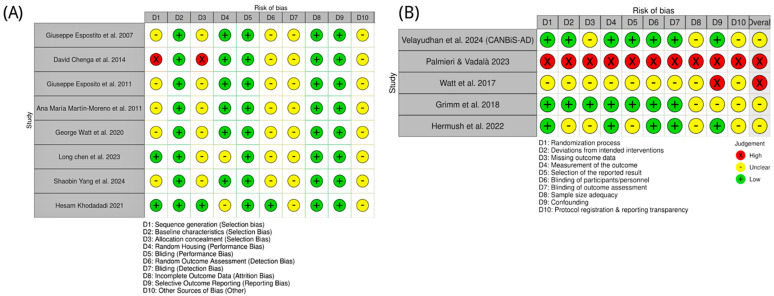
Risk of bias assessment for included studies on CBD in AD and related models. (**A**) Preclinical studies, with bias domains D1–D10 referring to selection, performance, detection, attrition, reporting, and other biases as defined in the key [71,72,82,83,84,85,86,87]. (**B**) Clinical studies, with bias domains D1–D10 covering randomization, intervention deviations, missing outcome data, outcome measurement, reporting, blinding, sample size adequacy, confounding, and protocol transparency [88,89,90,91,92]. Green circles indicate low risk of bias; yellow circles indicate unclear risk, and red circles indicate high risk.

Animal studies of CBD in AD were appraised with SYRCLE’s Risk of Bias tool. Most reports provided insufficient details on sequence generation and allocation concealment, yielding “unclear” risk for these domains. One study (Cheng et al. [72]) used a quasi-randomized design and was judged high risk for both domains. By contrast, Chen et al. [85] and Khodadadi et al. [87] explicitly described random allocation and were rated low risk for sequence generation. Baseline characteristics were consistently low risk, indicating good group balance. Randomized housing and performance bias were generally low risk, reflecting adequate environmental control and personnel blinding. However, outcome assessor blinding, detection bias, and “other bias” were commonly unclear due to sparse methodological reporting. Completeness of outcome data and selective reporting were uniformly low risk. Overall, preclinical studies showed low-to-moderate risk of bias, with key gaps in randomization reporting (Figure 5).

### 4.2. Risk of Bias: Clinical Studies

Clinical evidence was evaluated with the Cochrane RoB 2.0 tool. The CANBiS-AD trial (Velayudhan et al. [88]) was low risk across most domains, consistent with a randomized, double-blind, placebo-controlled design. Hermush et al. [92] also performed well for randomization, blinding, and outcomes, with some uncertainty regarding sample size and protocol transparency. Grimm et al. [91] were methodologically sound but assessed healthy volunteers, limiting generalizability to AD. The study of Watt et al. [81] lacked a specification of several key methods and was rated high risk. Palmieri & Vadalà’s [89] work employed an open-label design and was high risk across multiple domains. As summarized in Figure 5, clinical study quality varied: one high-risk study, two with some concerns, and two at low risk.

### 4.3. Meta-Analysis of Preclinical Trials: CBD and AD Neuroinflammatory Markers

**Figure 6:**
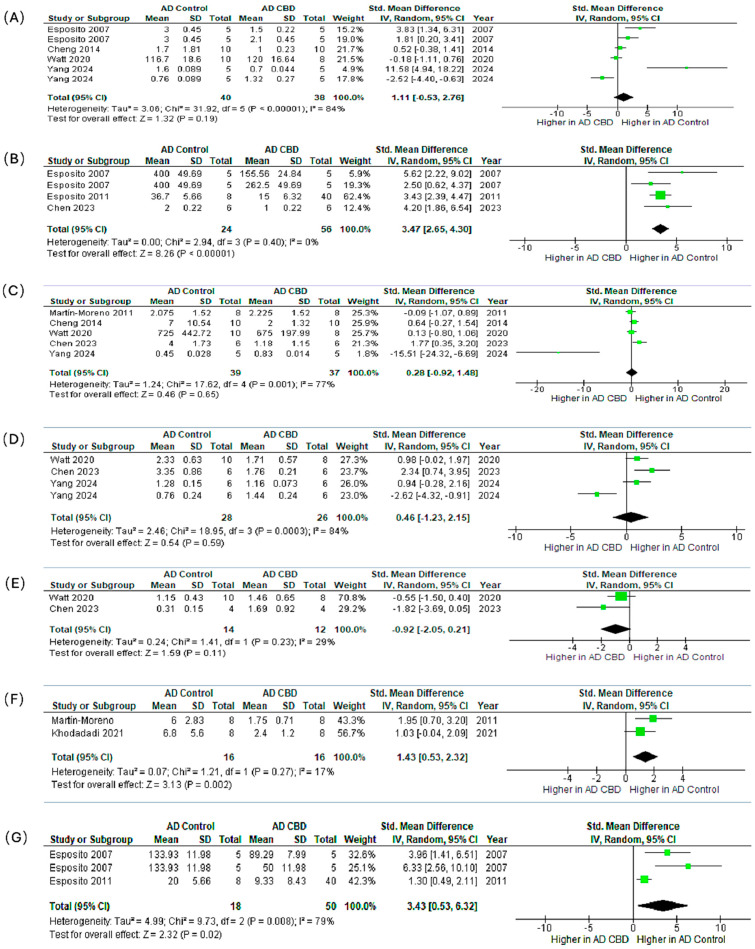
Meta-analysis of the effects of CBD in preclinical AD models on neuroinflammatory and neuroplasticity markers. The subfigures (**A**–**G**) represent the meta-analysis results for the following markers, respectively: (**A**) IL-1β [71,72,82,84,86,87], (**B**) GFAP [71,82,85], (**C**) TNF-α [72,83,84,85,86], (**D**) IBA1 [84,85,86,87], (**E**) BDNF [84,85], (**F**) IL-6 [83,87], and (**G**) iNOS [71,82,87]. In the forest plots, the green squares represent the individual study SMD, scaled by study weight, with the horizontal lines marking the 95% CI. The large black diamond represents the overall pooled SMD and its 95% CI, while the central vertical line is the line of no effect (SMD = 0).

We synthesized preclinical outcomes across seven markers (IL-1β, GFAP, TNF-α, IBA1, BDNF, IL-6, iNOS) using SMDs (95% CI) and random-effects models (Figure 6A–G).

**IL-1β** (Figure 6A). The pooled effect modestly favored CBD but was not significant (SMD = 1.11, 95% CI: −0.53–2.76; *p* = 0.19; I^2^ = 84%). Esposito et al. [71] showed a significant reduction; other studies reported smaller, non-significant changes.

**GFAP** (Figure 6B). CBD robustly reduced astrocytic activation with consistent effects (SMD = 3.47, 2.65–4.30; *p* < 0.00001; I^2^ = 0%), indicating attenuation of reactive astrogliosis.

**TNF-α** (Figure 6C). The pooled estimate favored CBD but was non-significant (SMD = 0.28, −0.92–1.48; *p* = 0.65; I^2^ = 77%), with study-level results ranging from modest decreases to no effect.

**IBA1** (Figure 6D). Effects on microglial marker IBA1 were heterogeneous and non-significant overall (SMD = 0.46, −1.23–2.15; *p* = 0.59; I^2^ = 84%), reflecting mixed increases and decreases across experiments.

**BDNF** (Figure 6E). Direction of effect varied across studies and did not reach significance (SMD = −0.92, −2.05–0.21; *p* = 0.11), suggesting context- or dose-dependent modulation of neurotrophic signaling.

**IL-6** (Figure 6F). CBD significantly lowered IL-6 with low heterogeneity (SMD = 1.43, 0.53–2.32; *p* = 0.002; I^2^ = 17%), supporting downstream dampening of pro-inflammatory cytokine cascades.

**iNOS** (Figure 6G). CBD significantly reduced iNOS (SMD = 3.43, 0.53–6.32; *p* = 0.02; I^2^ = 79%), consistent with suppression of nitric-oxide–mediated neuroinflammation.

**Synthesis.** Across all measured markers, the most statistically consistent and biologically relevant anti-inflammatory effects were observed for the astrocytic marker GFAP (*p* < 0.00001), the pro-inflammatory cytokine IL-6 (*p* = 0.002), and the inflammatory enzyme iNOS (*p* = 0.02). Non-significant or variable findings for IL-1β, TNF-α, IBA1, and BDNF were accompanied by substantial heterogeneity (I^2^ up to 84%). Likely sources include divergent CBD doses (0.5–20 mg/kg), treatment durations (7 days–8 weeks), animal models, assay methods, and disease stage at initiation. Collectively, these preclinical data support that CBD exerts reproducible astrocyte-modulating and pro-inflammatory cytokine-suppressive effects in AD models. Conversely, the inconsistent findings for microglial activation and neurotrophic signaling warrant future targeted, dose-ranging studies under harmonized protocols.

Forest plots show the standardized mean difference with 95% confidence intervals (CI) comparing CBD-treated groups versus AD controls. Each panel represents pooled effects on a specific marker: (A) IL-1β, (B) GFAP, (C) TNF-α, (D) IBA1, (E) BDNF, (F) IL-6, and (G) iNOS. Panels (B), (F), and (G) show significant reductions in GFAP, IL-6, and iNOS expression, respectively, suggesting CBD’s potential role in attenuating reactive gliosis and neuroinflammation. The remaining markers (IL-1β, TNF-α, IBA1, BDNF) show non-significant trends toward reduction. With varying degrees of heterogeneity, potentially reflecting differences in dosage, treatment duration, and model characteristics. These results support the anti-inflammatory and neuroprotective potential of CBD in AD pathophysiology.

### 4.4. Meta-Analysis of Clinical Outcomes: Behavior, Agitation, Adverse Events, and Caregiver Distress

**Figure 7:**
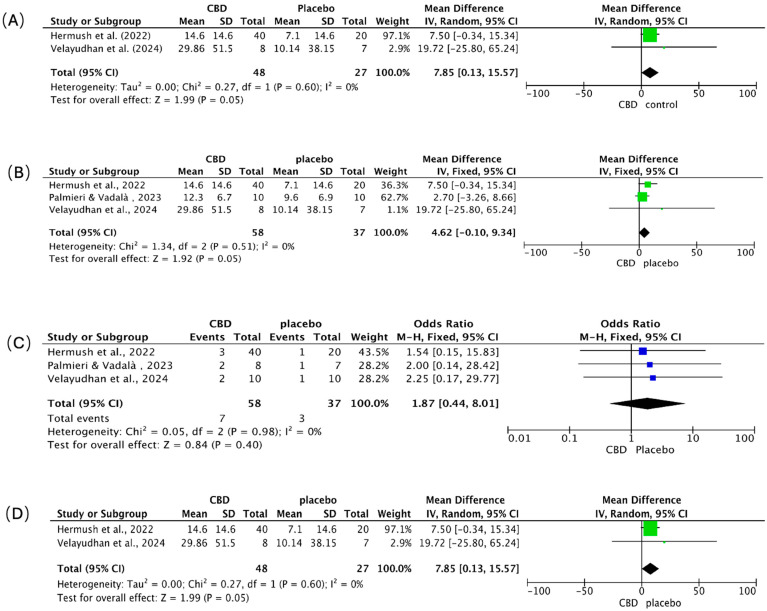
Meta-analysis of clinical trials assessing the effects of CBD in patients with AD. Forest plots depict the pooled effects of CBD treatment compared with placebo across four outcome domains: (**A**) overall behavioral symptoms [88,92], (**B**) agitation [88,89,92], (**C**) adverse events [88,89,92], and (**D**) caregiver distress [88,92]. The green squares represent the individual study SMD, and the blue squares represent the individual study Odds Ratio (OR); both are scaled by study weight, with the horizontal lines marking the 95% CI. The large black diamond represents the overall pooled SMD or OR and its 95% CI, while the central vertical line is the line of no effect (e.g., SMD = 0 or OR = 1.0).

We pooled continuous outcomes as mean differences (MD) and adverse events as odds ratios (OR), each with 95% CIs (Figure 7A–D). Positive MDs indicate greater symptom or burden reduction with CBD. Across panels A, B, and D, heterogeneity was absent (I^2^ = 0%).

**Behavioral symptoms** (Figure 7A). CBD favored improvement over placebo (pooled MD = 7.85; 95% CI 0.13–15.57; *p* = 0.05). Hermush et al. [92] showed a 7.5-point benefit (95% CI −0.34 to 15.34); Velayudhan et al. [88] reported a larger, imprecise effect (MD = 19.72; 95% CI −25.80 to 65.24), reflecting a small sample size.

**Agitation** (Figure 7B). Results numerically favored CBD (pooled MD = 4.62; 95% CI −0.10 to 9.34; *p* = 0.05), with consistent direction across studies. Hermush et al. [92] observed a 7.50-point reduction (95% CI −0.34 to 15.34); Palmieri & Vadalà [89] reported a smaller change (MD = 2.70; 95% CI −3.26 to 8.66) using a THC: CBD formulation; Velayudhan et al. [88] again showed the largest but least precise estimate (MD = 19.72; 95% CI −25.80 to 65.24).

**Adverse events** (Figure 7C). CBD did not increase overall adverse events versus placebo (pooled OR = 1.87; 95% CI 0.44–8.01; *p* = 0.40). Events were infrequent and mild (e.g., transient dizziness, somnolence, dry mouth, gastrointestinal discomfort) across all trials; no severe events, cognitive worsening, or clinically meaningful drug–drug interactions were attributed to CBD over short- to mid-term follow-up.

**Caregiver distress** (Figure 7D). CBD was associated with reduced caregiver burden (pooled MD = 7.85; 95% CI 0.13–15.57; *p* = 0.05). Hermush et al. [92] showed a marked decline in NPI caregiver distress scores versus placebo; while Velayudhan et al. [88] reported a numerically larger reduction, its precision was limited, a constraint shared by many studies in this area due to small sample sizes.

**Synthesis.** Clinical signals consistently favored CBD for behavioral symptoms, agitation, and caregiver distress, with borderline statistical significance and zero observed heterogeneity, but precision was limited by small samples and, in one study, the use of a THC: CBD combination. Tolerability appeared favorable. Larger, rigorously blinded, CBD-only trials with standardized endpoints are needed to confirm efficacy and better define the safety profile.

## 5. Discussion of the Results of the Integrated Meta-Analysis

### 5.1. Overview of the Results of the Integrated Meta-Analysis

#### 5.1.1. Neuroinflammatory Modulation of CBD in Animal Models of AD: Results of Meta-Analysis

Across preclinical AD models, CBD generally demonstrates an anti-inflammatory and neuroprotective profile; however, the direction and magnitude of effects are highly dependent on baseline neuroinflammation levels, CBD exposure pattern, host genotype, and the predominant receptor pathways engaged within a specific model. This variability is especially evident for TNF-α and IL-1β. For TNF-α, younger or early-stage animals often exhibit low inflammatory tone, limiting room for observable change; genotype and the choice of outcome modality add further dispersion. Mechanistically, CB2-linked effects can diverge from responses seen when TRPV2 or other non-CB2 routes dominate, and transcript protein discordance can confound interpretation, together suggesting that CBD may curb escalation rather than uniformly depress TNF-α across contexts [71,72,75,77,78,79,80,81,84,86,93,94]. A similar pattern emerges for IL-1β: CBD frequently dampens microglial and astrocytic drivers of this cytokine via CB2 and allied signaling, but responses can bifurcate by exposure duration and receptor engagement, with acute regimens aligning more consistently with attenuation and chronic paradigms more sensitive to pathway switching toward TRPV2 [43,72,73,84,86]. These observations argue for designs that prespecify inflammatory thresholds at treatment start, map receptor dominance over time, and privilege protein level readouts when feasible.

Astrocytic and microglial markers further illustrate CBD’s context dependence while clarifying plausible mechanisms. When gliosis is prominent, CBD is often associated with reduced GFAP, with PPARγ repeatedly implicated as a key mediator; antagonism at this receptor blunts CBD’s neuroprotective signature and links astrocyte modulation to preservation of neuronal integrity [71,73]. Microglial activation indexed by IBA1 shows a time and pathway-contingent pattern: reductions are more evident with acute exposure, whereas some chronic conditions, especially those engaging TRPV2/Akt (Protein Kinase B), report increases, likely reflecting differences in baseline microglial tone, genotype, and the balance between homeostatic surveillance and pro-inflammatory activation [71,84,86]. This underscores the value of multimodal microglial readouts (morphology, cytokines, phagocytic markers) to avoid over-interpreting a single marker.

Neurotrophic signaling remains inconclusive. Evidence for CBD’s influence on BDNF is limited and region-dependent, with inconsistencies across assay modalities. Given CBD’s pleiotropic nature, any BDNF changes are likely secondary to shifts in glial state and cytokine milieu, necessitating coordinated sampling that pairs behavioral phenotypes with trophic factors and cell-specific markers [86,94]. By contrast, IL-6 and iNOS findings more consistently align with down-modulation and attenuation, respectively, albeit with important moderators age, biological sex, and CBD formulation that must be prospectively stratified and connected to exposure via pharmacokinetics/pharmacodynamics (PK/PD) sampling [71,73,87]. Mechanistically, the recurring involvement of CB2 and PPARγ alongside contributions from TRPV2 and Wnt/β-catenin cautions against single-pathway models and supports integrated multi-target frameworks that can explain divergent outcomes across designs [71,73].

Taken together, preclinical heterogeneity is mechanistically informative rather than merely inconvenient. Future work should leverage it: enrich for animals with demonstrable inflammatory tone at baseline; separate study arms by hypothesized receptor axis (CB2, PPARγ, TRPV2); compare acute and chronic exposure with predefined criteria for pathway related paradoxical responses; and prioritize analysis plans that integrate protein level cytokines, glial state markers, and region specific histopathology to link target engagement with tissue level protection [71,72,84,86].

#### 5.1.2. Clinical Efficacy Study of CBD in Patients with AD: Results of a Meta-Analysis

Early clinical investigations—spanning randomized and observational designs converge on potential benefits for agitation and nocturnal symptoms in AD, with generally acceptable short- to mid-term tolerability; however, heterogeneity in samples, dosing regimens, CBD formulations, treatment duration, and outcome measures constrains certainty and limits formal synthesis [87,88,89,91]. Within this heterogeneity, a coherent narrative emerges. Behavioral improvements tend to cluster in domains governed by arousal and sleep–wake regulation, consistent with a dual mechanism that integrates anti-inflammatory effects with neuromodulation across 5-hydroxytryptamine receptor 1A, GABAergic, and endocannabinoid circuits [88,89]. Signals for reduced agitation often co-occur with better sleep consolidation and fewer hostile or impulsive behaviors, suggesting network-level modulation rather than isolated symptom effects; designs enriched for agitation-predominant phenotypes and augmented with digital actigraphy, or caregiver-triggered event capture may be better positioned to detect change [88,89,92].

Safety findings are broadly reassuring reported adverse events are typically mild and transient, with no consistent indication of accelerated cognitive decline. That said, older adults with polypharmacy require proactive surveillance for hepatic laboratory changes and drug–drug interactions, especially as trials extend exposure or escalate dose [88,92]. Observational studies add ecological validity but often lack controls or involve mixed cannabinoid preparations, complicating attribution and underscoring the need for receptor-informed, biomarker-guided trials that can parse CBD’s contribution within complex regimens [89,91]. Overall, the literature supports cautious optimism and a shift toward designs that purposely align behavioral endpoints with mechanistic biomarkers to determine who benefits, by which pathway, and under what exposure conditions [88,89,92].

### 5.2. Systematic Comparison and Translational Implications of Preclinical and Clinical Findings

Viewed together, bench and bedside findings are more complementary than contradictory. In controlled preclinical systems, CBD reliably maps to anti-inflammatory and neuroprotective mechanisms tempering microglia, modulating astrocytes, and reducing cytokine output via CB2 and PPARγ, with contributions from TRPV2 and Wnt/β-catenin [71,73,74]. Clinical contexts introduce real-world variability, comorbidities, concomitant medications, heterogeneous disease staging, and neuropsychiatric phenotypes, and diverse instruments that can dilute mechanistic signals and compress observable benefits [88,89]. The gap thus reflects differences in aim and constraint rather than a failure of pharmacology.

A streamlined translational agenda follows naturally. First, adopt mechanism-anchored clinical designs that prespecify pathway targets (CB2 vs. PPARγ vs. TRPV2) and embed target-engagement biomarkers, microglial positron emission tomography where feasible, fluid cytokine panels, and astrocytic markers paired with behavioral and functional endpoints to link biology to symptoms [71,73,74]. Second, optimize exposure by comparing acute, sub-chronic, and chronic regimens with explicit titration and adaptive rules for paradoxical responses; incorporate therapeutic drug monitoring where feasible to bridge PK with pharmacodynamics [86,87]. Third, differentiate isolate from full-spectrum CBD, characterize cannabinoid/terpenoid profiles, and model interactions with common AD and psychotropic medications; age- and sex-aware dosing with proactive hepatic surveillance should be standard [87,88]. Fourth, enrich cohorts for biomarker-positive neuroinflammation or agitation-predominant phenotypes while avoiding inflammatory floor/ceiling effects, and use staging frameworks that integrate cognitive and neuropsychiatric dimensions [88,89]. Fifth, increase endpoint sensitivity by pairing caregiver-reported scales with digital actigraphy and passive sensing to capture sleep, agitation cycles, and activity patterns, while including intermediate biological readouts that can distinguish symptomatic relief from disease modification [88,92]. Finally, harmonize safety monitoring across studies and systematically capture falls, sedation, and orthostatic symptoms given geriatric vulnerability, and ensure equitable, pragmatic recruitment with supports that enhance adherence and generalizability [91,92].

### 5.3. Limitation

Interpretation of these findings is tempered by several factors. Most preclinical experiments and clinical trials enrolled modest sample sizes, thereby limiting statistical power, precision, and generalizability; small studies also raise the risk of unstable or inflated effect estimates [73,88,92]. Beyond size, substantial design heterogeneity was evident. Animal studies differed in sex, body weight, disease induction, CBD dose and formulation, route, and treatment duration. Clinical cohorts varied in age, disease stage, concomitant medications, baseline severity, and inclusion criteria. This inherent variability undermines comparability, reproducibility, and external validity.

A further challenge is the divergence in research emphasis. Preclinical work prioritizes molecular mechanisms (e.g., NF-κB, PPARγ, NLRP3) and inflammatory readouts, whereas clinical trials focus on symptoms and quality-of-life outcomes (agitation, cognition, caregiver burden). Critically, human mechanistic evidence specific to AD remains sparse; many mechanistic studies were conducted in other conditions or healthy volunteers, widening the bench-to-bedside gap [73].

Study conduct and reporting were also uneven. Many preclinical reports lacked essential details on randomization, allocation concealment, and blinding. Although some clinical trials used randomized, controlled designs, they differed in dose, formulation, route, and duration, coupled with typically short follow-up periods, limited robust conclusions regarding sustained efficacy and long-term safety. This small clinical evidence base constrained the meta-analytic scope, curtailing informative subgroup and sensitivity analyses. Safety monitoring often emphasized short-term tolerability, with limited systematic assessment of liver function, drug–drug interactions, or long-term neuroadaptation, critical considerations for older adults with multimorbidity [88,92].

These limitations likely contributed to the high between-study heterogeneity observed, complicating inference and reducing confidence in pooled estimates. While random effects models and subgroup analyses were used to mitigate variability, statistical adjustments cannot fully compensate for underlying methodological divergence [73,88,92].

## 6. Conclusions

CBD remains a biologically plausible, multi-pathway candidate for modulating neuroinflammation and behaviorally relevant circuits in AD. Preclinical signals are broadly convergent; clinical signals, especially for agitation and caregiver burden, are promising yet heterogeneous, reflecting population complexity and design variation rather than an absence of pharmacological activity. To translate this mechanistic promise, future research must prioritize biomarker-guided, receptor-informed, and exposure-optimized clinical trials with standardized safety surveillance, ultimately providing actionable guidance on patient stratification, therapeutic mechanism, and optimal treatment conditions [71,72,73,74,75,76,81,84,85,86,87,88,89,90,91,92,93,94,95].

## Figures and Tables

**Table 1:** Summary of eight selected preclinical studies investigating the effects of CBD and CBD-containing preparations on A and related neuroinflammation. The table outlines study titles and references, primary objectives, methodologies, main findings, limitations, and the proposed relationship between CBD and neuroinflammatory processes, including modulation of microglial activation and regulation of pro-inflammatory cytokines (e.g., IL-1β, IL-6, TNF-α).

Study Name and Reference	Study Objective	Methodology	Main Finding	Limitation	CBD & Neuroinflammation Relationship
Cannabidiol in vivo blunts β-amyloid-induced neuroinflammation. Esposito et al. [71]	Examine CBD’s effect on neuroinflammation in AD mouse models by measuring IL-1β, iNOS, and GFAP expression.	Cross-sectional study, 5 mice per group, intraperitoneal CBD (2.5–10 mg/kg) for 7 days, immunofluorescence analysis.	CBD reduced IL-1β and iNOS, indicating anti-inflammatory effects in AD models.	Short treatment duration; does not assess long-term effects.	CBD reduces astrocytic and microglial activation, lowering IL-1β and iNOS.
Long-Term Cannabidiol Treatment Prevents Social Recognition Deficits. Cheng et al. [72]	Assess long-term effects of CBD on social recognition of memory and inflammatory markers IL-1β and TNF-α.	Longitudinal study, 10 mice per group, oral CBD (20 mg/kg) for 8 months, quantitative PCR for IL-1β and TNF-α.	CBD improved social recognition and slightly reduced neuroinflammation (IL-1β, TNF-α).	Limited to one behavioral measure; lacks molecular mechanism validation.	CBD mitigates the inflammatory effects of IL-1β and TNF-α in social recognition.
Cannabidiol Reduces Ab-Induced Neuroinflammation via PPARγ. Esposito et al. [82]	Investigate CBD’s role in reducing GFAP and iNOS via PPARγ activation in AD models.	Cross-sectional study, 40 mice, intraperitoneal CBD (10 mg/kg) for 15 days, Nissl staining, and densitometry.	CBD reduced neuroinflammation via PPARγ activation, decreasing GFAP and iNOS.	PPARγ involvement was inferred but not directly validated.	PPARγ activation by CBD suppresses GFAP and iNOS, reducing neuroinflammation.
Cannabidiol and Other Cannabinoids Reduce Microglial Activation. Martín-Moreno et al. [83]	Analyze CBD’s impact on microglial activation and pro-inflammatory cytokines in vivo.	Cross-sectional study, 8 mice per group, intraperitoneal CBD (20 mg/kg) for 4 weeks, qPCR for TNF-α and IL-6.	CBD reduced microglial activation and decreased TNF-α and IL-6 expression.	Small sample size; lacks long-term follow-up.	CBD downregulates microglial pro-inflammatory response, reducing TNF-α and IL-6.
Chronic Treatment with Cannabidiol Improves Cognition in AD Models. Watt et al. [84]	Evaluate CBD’s ability to improve cognition and modulate TNF-α, IL-1β, IBA1, and BDNF in AD models.	Cross-sectional study, 8–10 mice per group, intraperitoneal CBD (50 mg/kg) for 3 weeks, ELISA, and Western blot.	CBD improved cognition and reduced TNF-α, IBA1, and BDNF levels but increased IL-1β.	CBD increased levels of IL-1β, complicates interpretation.	CBD modulates cytokine levels, improving cognitive outcomes in AD.
Assessing Cannabidiol as a Therapeutic Agent in Alzheimer’s Disease. Chen et al. [85]	Study CBD’s effects on GFAP, TNF-α, IBA1, and BDNF levels in AD models.	Cross-sectional study, 6–10 mice per group, intragastric CBD (25 mg/kg) for 13 days, immunofluorescence analysis.	CBD lowered GFAP, TNF-α, and IBA1 levels while increasing BDNF, enhancing cognitive function.	Small sample size; requires a dose-dependent study.	CBD alters neuroinflammatory marker expression, promoting cognitive benefits.
Tyrosine phosphorylation and palmitoylation of transient receptor potential cation channel subfamily V member 2 (TRPV2) tune microglial phagocytosis. Yang et al. [86]	Assess the duration-dependent effect of CBD on neuroinflammation and microglial phagocytosis.	Cross-sectional study, 6 mice per group, intraperitoneal CBD (10 mg/kg) for 1–3 weeks, Western blot and qPCR.	Short-term CBD reduced neuroinflammation, but long-term CBD increased neuroinflammatory markers.	Long-term effects of CBD on neuroinflammation require further study.	CBD’s effects on neuroinflammation are duration-dependent, requiring careful dosing.
Cannabidiol Ameliorates Cognitive Function via IL-33 and TREM2 Upregulation. Khodadadi et al. [87]	Investigate CBD’s impact on IL-6 levels and cognitive function in an AD murine model.	Cross-sectional study, 6–10 mice per group, intraperitoneal CBD (10 mg/kg) every other day for 2 weeks, flow cytometry.	CBD significantly reduced IL-6 and improved cognitive function.	Limited to IL-6; lacks other neuroinflammatory markers.	CBD significantly reduces IL-6 levels, improving neuroprotection in AD models.

**Table 2:** Summary of selected 5 clinical trials on CBD or CBD-containing preparations in AD and dementia. The table outlines study details, key findings, and limitations, highlighting proposed mechanisms such as microglial modulation and reduction in pro-inflammatory cytokines (e.g., IL-1β, IL-6, TNF-α), with these pathways functioning.

Study Name and Reference	Study Objective	Methodology	Main Finding	Limitation	CBD & Neuroinflammation Relationship
Cannabidiol for Behavioral Symptoms in Alzheimer’s Disease (CANBiS-AD)—Randomized Controlled Trial on Agitation and Dementia Symptoms. Velayudhan et al. [88]	To test CBD efficacy for behavioral symptoms in Alzheimer’s disease patients.	Randomized, double-blind, placebo-controlled trial; 120 participants; 12-week oral CBD (up to 300 mg/day).	Preliminary data suggest that CBD improved agitation and caregiver distress.	Small sample size and short follow-up period.	Likely works by reducing microglial activation and neuroinflammatory cytokines such as IL-1β and TNF-α.
Oral THC: CBD Cannabis Extract for Alzheimer’s Symptoms—Investigating Agitation and Weight Loss in AD Patients. Palmieri et al. [89]	To investigate mixed THC: CBD extract in treating agitation and weight loss in AD.	Open-label clinical trial; oral THC: CBD extract for 12 weeks; behavioral assessments and weight tracking.	Reported reduced agitation and slight weight stabilization.	THC confounds CBD-specific effects; open-label design limits rigor.	Neuroinflammation modulation may be partially attributable to CBD, but THC’s role cannot be excluded.
In vivo Evidence for Therapeutic Properties of Cannabidiol for Alzheimer’s Disease. Watt et al. [90]	To explore CBD’s anti-inflammatory effects in multiple sclerosis (MS) models.	Preclinical study using EAE mice and human MS patient samples. Measured TNF-α, IL-1β, and microglial activation.	CBD reduced neuroinflammation by suppressing microglial activation and TNF-α/IL-1β levels in both mice and humans.	Limited human data. Focused on preclinical evidence.	CBD inhibits TLR4/NF-κB signaling, reducing pro-inflammatory cytokines in MS.
Probing the endocannabinoid system for healthy volunteers. Grimm et al. [91]	Probing the endocannabinoid system in healthy volunteers	Clinical study in healthy volunteers; CBD administration; resting-state fMRI connectivity analysis.	CBD altered fronto-striatal resting-state connectivity in healthy volunteers.	Study on healthy volunteers; not Alzheimer-specific.	Not directly related to Alzheimer neuroinflammation; general brain connectivity finding.
Effects of Rich Cannabidiol Oil on Behavioral Disturbances in Dementia—Placebo-Controlled RCT Evaluating Agitation Reduction. Hermush et al. [92]	To assess CBD oil’s impact on behavioral symptoms in dementia.	Placebo-controlled RCT; CBD oil (30% concentration) given for 6 weeks to dementia patients.	Reduced agitation and caregiver distress scores	Small sample size, short intervention duration.	CBD likely reduced microglial activity and pro-inflammatory cytokines (IL-6, TNF-α).

## Data Availability

The data that support the findings of this meta-analysis are publicly available and were derived entirely from the published studies cited in the reference list of this article. No new data were generated.
